# Supplementary material for: mRNA booster vaccination protects aged mice against the SARS-CoV-2 Omicron variant
Source: Commun Biol. 2022 Aug 6;5:790. doi: 10.1038/s42003-022-03765-3 (PMC9357006; doi:10.1038/s42003-022-03765-3)
Supplement: Supplementary file 2 — Supplementary Material [file 42003_2022_3765_MOESM2_ESM.pdf]

Supplementary Material for

"mRNA booster vaccination protects aged mice against the SARS-CoV-2 Omicron variant"

Etsuro Nanishi[1,2†], Marisa E. McGrath[3†], Timothy R. O'Meara[1], Soumik Barman[1,2], Jingyou Yu[4], Huahua Wan[4], Carly A. Dillen[3], Manisha Menon[1], Hyuk-Soo Seo[5,6], Kijun Song[5], Andrew Z Xu[5], Luke Sebastian[5], Byron Brook[1,2], Anna-Nicole Bosco[1], Francesco Borriello[1,2,7,ϕ], Robert K. Ernst[8], Dan H. Barouch[4], Sirano Dhe-Paganon[5,6], Ofer Levy[1,2,9††], Matthew B. Frieman[3††], David J. Dowling[1,2††]

1) *Precision Vaccines Program*, Division of Infectious Diseases, Boston Children's Hospital, Boston, MA, USA.
2) Department of Pediatrics, Harvard Medical School, Boston, MA, USA.
3) Department of Microbiology and Immunology, The Center for Pathogen Research, University of Maryland School of Medicine, Baltimore, MD, USA.
4) Center for Virology and Vaccine Research, Beth Israel Deaconess Medical Center, Harvard Medical School, Boston, MA, USA.
5) Department of Cancer Biology, Dana-Farber Cancer Institute, Boston, MA, USA.
6) Department of Biological Chemistry and Molecular Pharmacology, Harvard Medical School, Boston, MA, USA.
7) Division of Immunology, Boston Children's Hospital, Boston, MA, USA.
8) Department of Microbial Pathogenesis, University of Maryland School of Dentistry, Baltimore, MD, USA.
9) Broad Institute of MIT & Harvard, Cambridge, MA, USA.
ϕ Present address: Generate Biomedicines, Cambridge, MA, USA.

†These authors contributed equally to this manuscript.
††These authors jointly supervised this work.

***Corresponding author**: David J Dowling, *Precision Vaccines Program*, Division of Infectious Diseases, Boston Children's Hospital; Harvard Medical School, 4 Blackfan Circle, Harvard Institutes of Medicine, Rm 842, Boston, MA 02115, USA. Tel: +1 617-919-6890. e-mail: david.dowling@childrens.harvard.edu

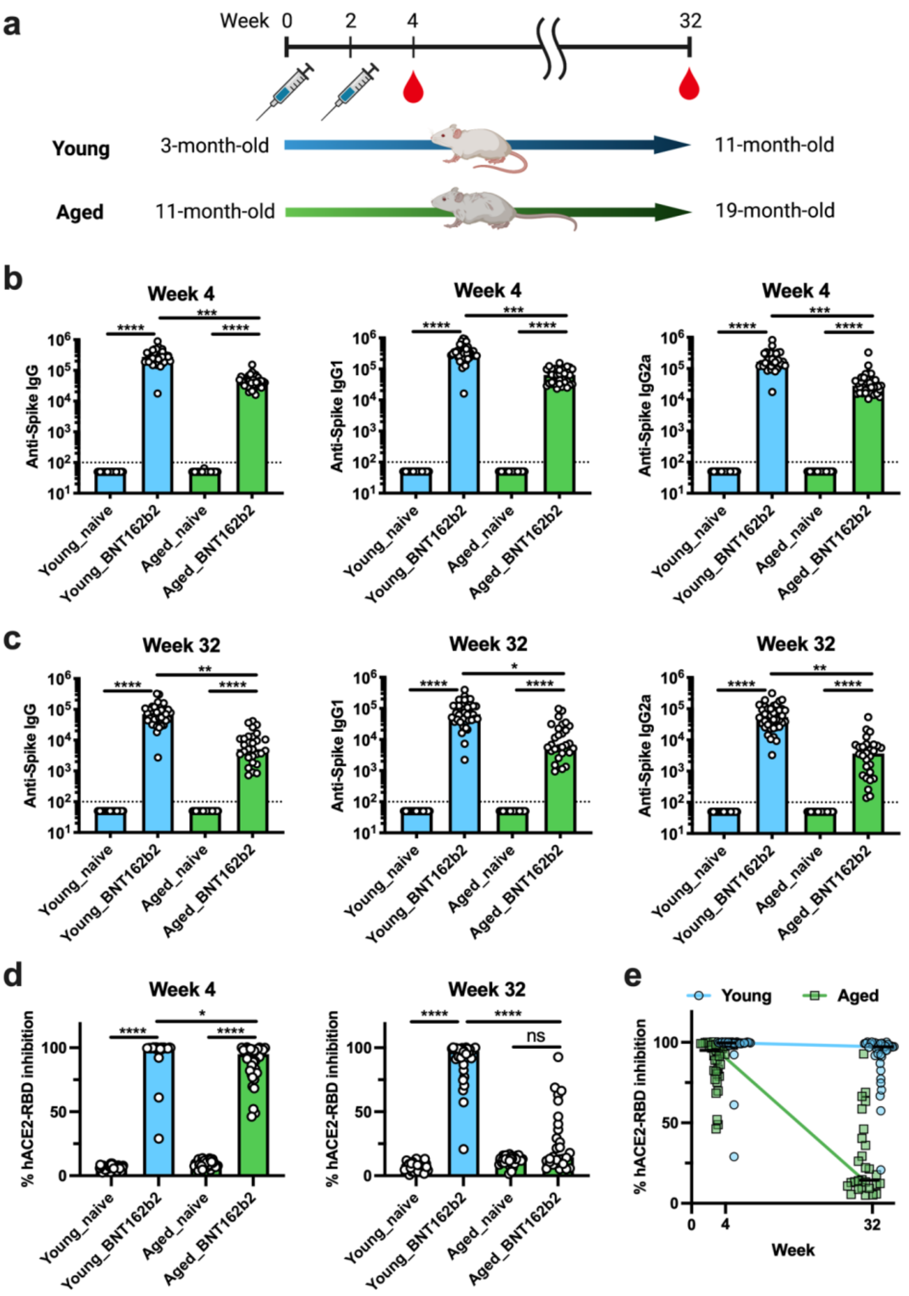

Supplementary Figure 1. Vaccine-induced humoral immune responses wane in 19-month-old mice at 32 weeks after 2-dose mRNA BNT162b2 immunization

Three- and 11-month-old BALB/c mice were immunized with 1 µg of mRNA BNT162b2 on weeks 0 and 2. Serum samples were collected at weeks 4 and 32 (**a**). Anti-spike IgG, IgG1, and IgG2a were assessed on week 4 (**b**) and week 32 (**c**), and (**d-e**) hACE2-RBD inhibition rate was assessed at both timepoints. N=39–40 per group for week 4 and N=28–40 per group for week 32. Results are presented as median. Each symbol represents an individual sample. Data were analyzed by Kruskal-Wallis test corrected for multiple comparisons. *P <0.05, **P <0.01, ***P <0.001, **** P <0.0001. Dashed lines represent lower limit of detection. The graphic in Supplementary Figure 1a was created with BioRender.com.

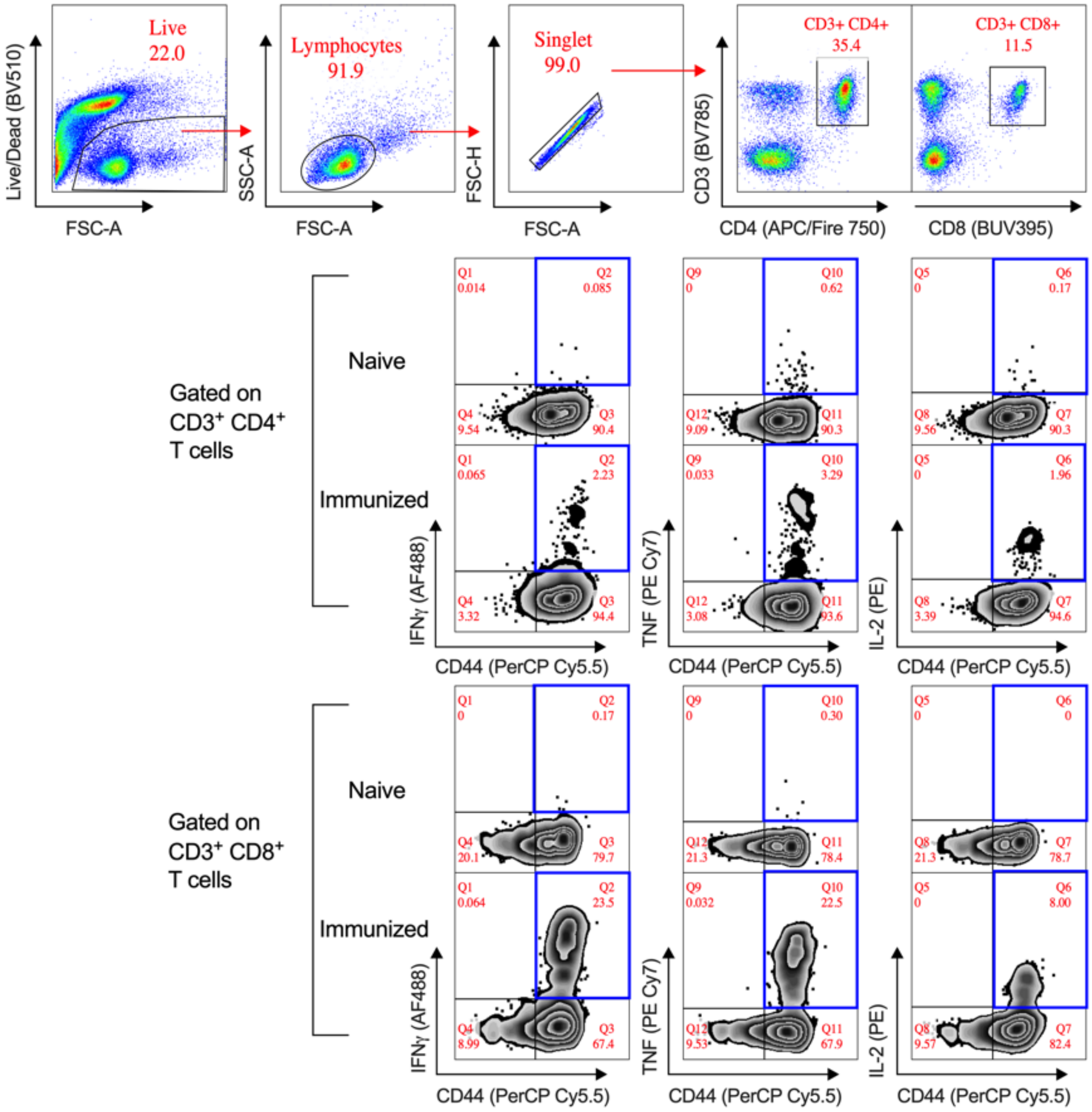

Supplementary Figure 2. ***Ex vivo*** **flow cytometry gating strategy post murine mRNA vaccination**

Representative flow cytometry plots showing the gating strategy applied to identify spike-specific CD4$^+$ and CD8$^+$ T cell responses to peptide pools post murine mRNA vaccination, representing wildtype SARS-CoV-2 (WT) or Omicron.

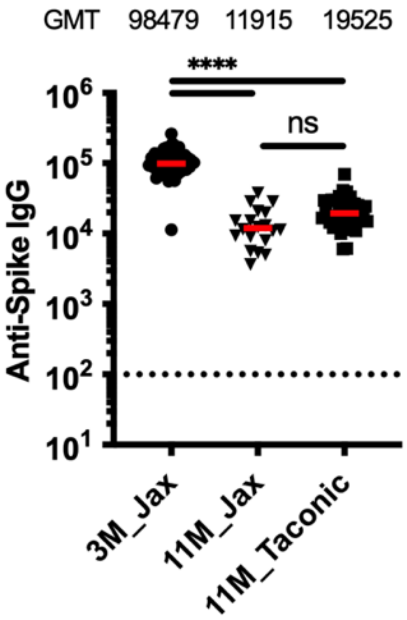

Supplementary Figure 3. 11-month-old BALBc mice demonstrate similar anti-spike IgG responses after primary mRNA vaccination series

3-month-old BALB/c mice acquired from the Jackson Laboratory (Jax) received primary mRNA vaccination series at either study week 0 at the age of 3-month ("3M_Jax") or study week 32 at the age of 11-month ("11M_Jax"). 11-month-old BALB/c mice acquired from Taconic and received primary vaccination series at study week 0 ("11M_Taconic"). Sera were collected 2 weeks after primary vaccination series and anti-spike IgG titers were determined. Each symbol represents an individual sample and red horizontal lines and numbers represents geometric mean titers (GMTs). Dashed lines represent lower limit of detection. N=40, 39 and 19 per group for "3M_Jax", "11M_Jax", and "11M_Taconic", respectively. Data were analyzed by Kruskal-Wallis test corrected for multiple comparisons. **** *P* <0.0001.
