# Supplementary material for: mRNA booster vaccination protects aged mice against the SARS-CoV-2 Omicron variant
Source: Commun Biol. 2022 Aug 6;5:790. doi: 10.1038/s42003-022-03765-3 (PMC9357006; doi:10.1038/s42003-022-03765-3)
Supplement: Supplementary file 5 — Reporting Summary [file 42003_2022_3765_MOESM5_ESM.pdf]

nature portfolio

Corresponding author(s): David Dowling

Last updated by author(s): Jun 23, 2022

Reporting Summary

Nature Portfolio wishes to improve the reproducibility of the work that we publish. This form provides structure for consistency and transparency in reporting. For further information on Nature Portfolio policies, see our Editorial Policies and the Editorial Policy Checklist.

Statistics

For all statistical analyses, confirm that the following items are present in the figure legend, table legend, main text, or Methods section.

n/a	Confirmed	
☐	☒	The exact sample size (*n*) for each experimental group/condition, given as a discrete number and unit of measurement
☐	☒	A statement on whether measurements were taken from distinct samples or whether the same sample was measured repeatedly
☐	☒	The statistical test(s) used AND whether they are one- or two-sided<br>*Only common tests should be described solely by name; describe more complex techniques in the Methods section.*
☒	☐	A description of all covariates tested
☐	☒	A description of any assumptions or corrections, such as tests of normality and adjustment for multiple comparisons
☐	☒	A full description of the statistical parameters including central tendency (e.g. means) or other basic estimates (e.g. regression coefficient) AND variation (e.g. standard deviation) or associated estimates of uncertainty (e.g. confidence intervals)
☐	☒	For null hypothesis testing, the test statistic (e.g. *F*, *t*, *r*) with confidence intervals, effect sizes, degrees of freedom and *P* value noted<br>*Give P values as exact values whenever suitable.*
☒	☐	For Bayesian analysis, information on the choice of priors and Markov chain Monte Carlo settings
☒	☐	For hierarchical and complex designs, identification of the appropriate level for tests and full reporting of outcomes
☒	☐	Estimates of effect sizes (e.g. Cohen's *d*, Pearson's *r*), indicating how they were calculated

Our web collection on statistics for biologists contains articles on many of the points above.

Software and code

Policy information about availability of computer code

Data collection	No unique software was used for data collection.
Data analysis	Data were analyzed with commercially available and open-source programs as stated in the methods section. Descriptive statistics were calculated with Prism 9.

For manuscripts utilizing custom algorithms or software that are central to the research but not yet described in published literature, software must be made available to editors and reviewers. We strongly encourage code deposition in a community repository (e.g. GitHub). See the Nature Portfolio guidelines for submitting code & software for further information.

Data

Policy information about availability of data

All manuscripts must include a data availability statement. This statement should provide the following information, where applicable:

- Accession codes, unique identifiers, or web links for publicly available datasets
- A description of any restrictions on data availability
- For clinical datasets or third party data, please ensure that the statement adheres to our policy

The authors declare that the data supporting the findings of this study are available within the main and supplemental figures. All data is available from the corresponding author upon reasonable request.

Field-specific reporting

Please select the one below that is the best fit for your research. If you are not sure, read the appropriate sections before making your selection.

☒ Life sciences ☐ Behavioural & social sciences ☐ Ecological, evolutionary & environmental sciences

For a reference copy of the document with all sections, see nature.com/documents/nr-reporting-summary-flat.pdf

Life sciences study design

All studies must disclose on these points even when the disclosure is negative.

Sample size	Mouse experiments aimed to include in total 20 mice per group and were from single experiments. Sample size and age criteria were chosen empirically based on the results of previous studies. Mice were randomly assigned to different treatment groups.
Data exclusions	No data were excluded.
Replication	Each binding study was performed one. Within binding studies we tested multiple dilutions to confirm the binding magnitude instead of relying on single data points. Neutralization assays have been validated to be reproducible.
Randomization	Mice were randomly distributed in groups.
Blinding	Neutralization assay was performed by laboratory independent from the discovery laboratory. No other data was supplied until after the assay was complete. For the remaining assays, blinding was not preformed. However, all experiments were carried out in an unbiased manner to prevent potential biases in the experimental groups. Statistics were not calculated until the study was complete and all data had been verified to be accurate.

Reporting for specific materials, systems and methods

We require information from authors about some types of materials, experimental systems and methods used in many studies. Here, indicate whether each material, system or method listed is relevant to your study. If you are not sure if a list item applies to your research, read the appropriate section before selecting a response.

Materials & experimental systems

n/a	Involved in the study	
☐	☒	Antibodies
☒	☐	Eukaryotic cell lines
☒	☐	Palaeontology and archaeology
☐	☒	Animals and other organisms
☒	☐	Human research participants
☒	☐	Clinical data
☒	☐	Dual use research of concern

Methods

n/a	Involved in the study	
☒	☐	ChIP-seq
☐	☒	Flow cytometry
☒	☐	MRI-based neuroimaging

Antibodies

Antibodies used	anti-mouse CD44 [IM7, PerCP-Cy5.5, BioLegend #103032, 1:160]<br>anti-mouse CD3 [17A2, Brilliant Violet 785, BioLegend #100232, 1:40]<br>anti-mouse CD4 [RM4-5, APC/Fire 750, BioLegend 100568, 1:160]<br>anti-mouse CD8 [53-6.7, Brilliant UltraViolet 395, BD #563786, 1:80]<br>anti-mouse IFNγ [XMG1.2, Alexa Fluor 488, BioLegend #505813, 1:160]<br>anti-mouse TNF [MP6-XT22, PE Cy7, BioLegend # 506324, 1:160]<br>anti-mouse IL-2 [JES6-5H4, PE, BioLegend # 503808, 1:40]
Validation	Commercial antibodies were used according to manufacturer's instructions and this is noted in the manuscript where appropriate.

Animals and other organisms

Policy information about studies involving animals; ARRIVE guidelines recommended for reporting animal research

Laboratory animals	Female, 3-month-old BALB/c mice were purchased from the Jackson Laboratory.<br>Female, 11-month-old BALB/c mice were purchased from Taconic Biosciences and used for aged mice experiments.
Wild animals	Not applicable

Field-collected samples	Not applicable
Ethics oversight	Mice were housed under specific pathogen-free conditions at Boston Children's Hospital. All the procedures were approved under the Institutional Animal Care and Use Committee (IACUC) and operated under the supervision of the Department of Animal Resources at Children's Hospital (Protocol number 19-02-3897R). At the University of Maryland School of Medicine, mice were housed in a biosafety level 3 facility for SARS-CoV-2 infections with all the procedures approved under the IACUC (Protocol #1120004).

Note that full information on the approval of the study protocol must also be provided in the manuscript.

Flow Cytometry

Plots

Confirm that:

☒ The axis labels state the marker and fluorochrome used (e.g. CD4-FITC).

☒ The axis scales are clearly visible. Include numbers along axes only for bottom left plot of group (a 'group' is an analysis of identical markers).

☒ All plots are contour plots with outliers or pseudocolor plots.

☒ A numerical value for number of cells or percentage (with statistics) is provided.

Methodology

Sample preparation	Mouse spleens were mechanically dissociated and filtered through a 70 µm cell strainer. After centrifugation, cells were treated with 1 mL ammonium-chloride-potassium lysis buffer for 2 min at RT. Cells were washed and plated in a 96-well U-bottom plate (2 x 106/well) and incubated overnight in RPMI 1640 supplemented with 10% heat-inactivated FBS, penicillin (100 U/ml), streptomycin (100 mg/ml), 2-mercaptoethanol (55 mM), non-essential amino acids (60 mM), HEPES (11 mM), and L-Glutamine (800 mM) (all Gibco).
Instrument	LSR II (BD) flow cytometer
Software	FlowJo v10.8.1 (FlowJo LLC)
Cell population abundance	No sorting was preformed.
Gating strategy	Gating strategies are supplied in supplemental figures.

☒ Tick this box to confirm that a figure exemplifying the gating strategy is provided in the Supplementary Information.
